# Complete *in vitro* oogenesis: retrospects and prospects

**DOI:** 10.1038/cdd.2017.134

**Published:** 2017-08-25

**Authors:** Jun-Jie Wang, Wei Ge, Jing-Cai Liu, Francesca Gioia Klinger, Paul W Dyce, Massimo De Felici, Wei Shen

**Affiliations:** 1 Institute of Reproductive Sciences, College of Life Sciences, Qingdao Agricultural University, Qingdao 266109, China; 2 Department of Biomedicine and Prevention, University of Rome ‘Tor Vergata’, Rome 00133, Italy; 3 Department of Animal Sciences, Auburn University, Auburn, AL 36849, USA

## Abstract

Precise control of mammalian oogenesis has been a traditional focus of reproductive and developmental biology research. Recently, new reports have introduced the possibility of obtaining functional gametes derived *in vitro* from stem cells. The potential to produce functional gametes from stem cells has exciting applications for regenerative medicine though still remains challenging. In mammalian females ovulation and fertilization is a privilege reserved for a small number of oocytes. In reality the vast majority of oocytes formed from primordial germ cells (PGCs) will undergo apoptosis, or other forms of cell death. Removal occurs during germ cell cyst breakdown and the establishment of the primordial follicle (PF) pool, during the long dormancy at the PF stage, or through follicular atresia prior to reaching the ovulatory stage. A way to solve this limitation could be to produce large numbers of oocytes, *in vitro,* from stem cells. However, to recapitulate mammalian oogenesis and produce fertilizable oocytes *in vitro* is a complex process involving several different cell types, precise follicular cell–oocyte reciprocal interactions, a variety of nutrients and combinations of cytokines, and precise growth factors and hormones depending on the developmental stage. In 2016, two papers published by Morohaku *et al.* and Hikabe *et al.* reported *in vitro* procedures that appear to reproduce efficiently these conditions allowing for the production, completely in a dish, of a relatively large number of oocytes that are fertilizable and capable of giving rise to viable offspring in the mouse. The present article offers a critical overview of these results as well as other previous work performed mainly in mouse attempting to reproduce oogenesis completely *in vitro* and considers some perspectives for the potential to adapt the methods to produce functional human oocytes.

## Facts

- Complete oogenesis *in vitro* and obtaining fertilizable oocytes are not only conducive to understanding the regulatory mechanisms of oogenesis, but also to improving the fecundity of mammalian females.
- Recently, reconstituting mouse oogenesis from endogenous PGCs or PGCLCs derived from ES or iPS cells completely *in vitro* have made significant breakthroughs.
- Among the three main steps involved in mammalian oogenesis *in vitro* (PGC induction, oocyte differentiation and growth and oocyte maturation/IVF), obtaining oocytes at the secondary follicle stage from PGCs or PGCLCs is the most challenging event during the oocyte differentiation process.
- Successful recapitulation of mouse oogenesis, under completely *in vitro* conditions, provides a valuable model for studying the mechanisms underlying mammalian oogenesis, particularly for human focused studies.

## Open Questions

- What’s responsible for the low efficiency of PGCLCs in producing viable oocytes compared with endogenous PGCs?
- Are there differences between offspring derived from endogenous PGCs and those derived from PGCLCs?
- Is the current system applicable to human stem cell derived oogenesis?
- Will the current system prove to be the ultimate strategy for female infertility treatments?

Due to their unique developmental and regulatory mechanisms, the majority of the mammalian oocyte resource is not fully utilized. For this reason, the generation of functional oocytes *in vitro* is not only an important target of reproductive research, aiming to identify the underlying regulatory mechanisms of oogenesis, but also a way to optimize the use of the female germ cell pool to improve present assisted reproductive technologies (ART) and to ameliorate female health.

**Figure 1:**
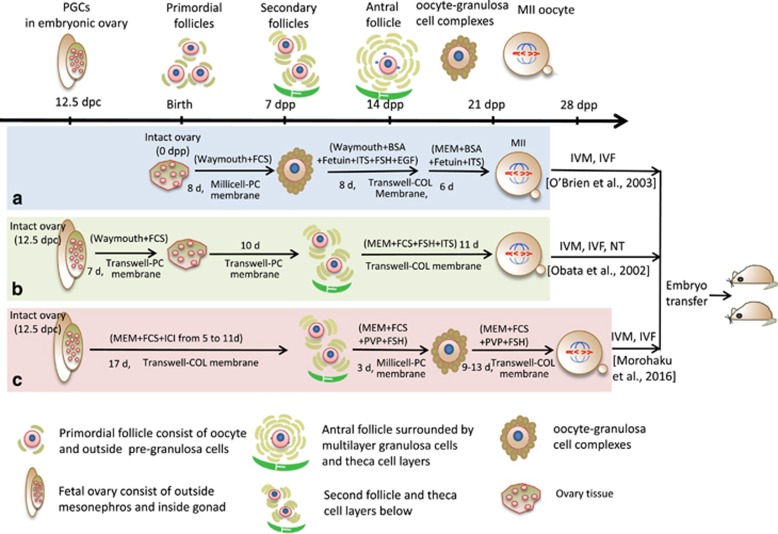
Schematic representation of the main stages of mouse oogenesis (upper drawing) and of the three *in vitro* culture methods of endogenous germ cells (medial drawings) capable of producing metaphase II (MII) oocytes that after *in vitro* maturation (IVM) and *in vitro* fertilization (IVF) or nuclear transfer (NT) generated live offspring; most essential components of the culture media are also reported. The upper drawing displays the process of oogenesis from 12.5 days post-coitum (dpc) and folliculogenesis, including the essential stages of embryonic primordial germ cells (PGCs) at 12.5 dpc, primordial follicles at birth, and the formation of secondary and antral follicles at 7 and 14 days post-partum (dpp), development of mature gametes as oocyte–granulosa cell complexes, oocyte meiosis and IVF. The medial figure shows the offspring through *in vitro* culture with 1 dpp ovary by O’Brien *et al.* (**a**) and fetal ovary at 12.5 dpc by Obata *et al.* (**b**) and Morohaku *et al.* (**c**) The lower panel shows the different cell types annotated

Oocyte *in vitro* maturation (IVM) and *in vitro* fertilization (IVF) technologies have been widely used in research and clinical medicine, while reproducing complete oogenesis *in vitro* and obtaining mature and fertilizable oocytes capable of giving rise to new individuals has remained elusive until very recently. As early as 1971, Odor and Blandau^1^ reported the first noteworthy results showing that it was possible to obtain a large number of growing mouse and human oocytes by culturing small pieces of fetal ovary in rose chambers for 3–5 weeks using a modified Eagle’s medium containing 10% heat inactivated horse serum. These oocytes were capable of completing the meiotic prophase with some reaching a diameter of 80–100 *μ*m, including the zona pellucida, within a multilaminar follicle. After these promising results, many other attempts were made to reconstitute the entire oogenesis process *in vitro* from follicular oocytes or primordial germ cells (PGCs) (Figure 1 and Table 1). Attempts included using isolated PGCs and culturing on cell monolayers or inside gonadal tissues (pieces of ovarian tissues, ovarian somatic cell aggregates and organotypic cultures). More recently, the possibility of obtaining PGCs from various types of pluripotent stem cells has provided a new cellular input and greatly increased the interest in such studies. However, only very recently two papers from the same research team have reported successful reconstitution of the complete mouse oogenesis process *in vitro* using the same protocol for all steps except the initial source of the PGCs (endogenous PGCs *vs* PGC-like cells (PGCLCs) derived from embryonic stem cells (ESCs) and induced pluripotent stem cells (iPSCs)).^2, 3^

In the present work, we review the main information and faults of experiments performed mainly in the mouse in the attempt to reconstruct oogenesis *in vitro* starting from PGCs that preceded these papers and made the extraordinary results achieved by these authors (Figure 1 and Table 1). Moreover, the main improvements introduced by the Morohaku *et al.*^2^ *in vitro* procedures allowing a robust number of oocytes to acquire complete meiotic maturation and the capacity to support embryo development after fertilization, will be discussed. Finally, some perspectives on the potential to transfer such results from mice to humans will be considered.

## Outlines of Mouse Oogenesis

Since the mouse is the species in which the papers by Morohaku *et al.* and Hikabe *et al.* were performed and most experimental studies of *in vitro* oogenesis were carried out, a brief outline of mouse oogenesis follows (Figure 1).

In the mouse embryo, a small number of epiblast cells (about 5–6) are induced to become the PGC precursors (around 6.0 days post-coitum, dpc) by various bone morphogenetic proteins (BMPs), in particular BMP4, secreted by the neighbouring extraembryonic tissues.^4^ These committed cells are then specified and destined to become PGCs while moving to, and allocated in, the allantois (7.5–8.5 dpc). After migration into the gonadal ridges (9.5–10.5 dpc), and some rounds of mitotic proliferation, PGCs differentiate into mitotic oogonia which then enter into meiosis (13.5–14.5 dpc) and become primary oocytes. Since PGC/oogonia mitosis often ends with incomplete cytokinesis, primary oocytes develop as clusters or nests of cells in which daughter cells are joined by intercellular bridges.^5^ Primary oocytes progress to meiotic prophase I and arrest at the diplotene stage around (start from 16.5 dpc) or early after birth. During this period more than one-third of the oocytes degenerate by apoptosis and/or autophagy.^6, 7^ At about 4 days post-partum (dpp), the majority of germ cells have disappeared^8^ and the surviving oocytes are individually encircled by pregranulosa cells forming a primordial follicle (PF). In the mouse ovary there is compelling evidence for the existence of two distinct classes of PFs; a first class form that are synchronously activated within a short time window around birth within the ovary medulla and a second class form in the cortex that are gradually activated in adulthood. It was calculated that a minimum of 23 and 47 days is needed for the first and the second classes of PFs, respectively, to reach the antral stage. The first wave of follicles exists in the ovaries for about 3 months and contributes to the onset of puberty and to early fertility. PFs in the ovarian cortex gradually replace the first wave of follicles providing fertility until the end of reproductive life.^9^

The PF pool represents the ‘ovarian reserve’ from which cohorts of follicles are continuously activated, from birth to menopause, to undergo extensive growth and development as primary and secondary follicles in a process termed folliculogenesis. Oocytes grow from 12–20 *μ*m in diameter in primordial and primary follicles, to 70 *μ*m in medium size preantral follicles and eventually to a fully grown size of approximately 80 *μ*m in large antral follicles. Growing oocytes start to synthesize the zona pellucida glycoproteins, accumulate RNAs and proteins, and are connected through gap junctions to surrounding granulosa cells, which supply them with small metabolites, such as energy substrates, nucleotides and amino acids. During the growing phase oocytes progressively acquire competence to resume meiosis, undergo fertilization and complete preimplantation development. Competence to resume meiosis is not achieved until late in oocyte development and appears correlated with the oocyte diameter (≥60 *μ*m), the appearance of a chromatin rim around the nucleolus and cytoskeletal reorganization.^10^ Shortly before ovulation, the meiotic cell cycle is reactivated in the oocytes inside the preovulatory antral follicles and another meiosis II arrest ensues (the metaphase II block); reactivation and completion of meiosis is then dependent upon fertilization. Depending on the strain, an adult female mouse produces from 4 up to 12 fertilizable oocytes every 4–5 days for about 7–8 months.

## Key Events For Mouse Oogenesis *In Vitro*

**Figure 2:**
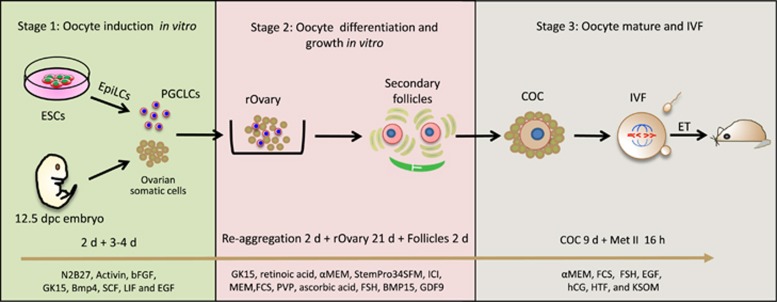
The main steps involved in the procedure of reconstructing the entire mouse oogenesis from exogenous stem cells *in vitro* (see Hikabe *et al.*^3^). This process was divided into three parts: oocyte induction *in vitro* (left panel), oocyte differentiation and growth *in vitro* (middle panel), oocyte maturation and IVF (right panel). Each panel consists of the experimental schematic and *in vitro* culture details involved in each process

Eppig and O'Brien^11^ applied a two-step culture method with isolated newborn mouse ovaries (PF stage) to generate live offspring in 1996. The initial step involved ovarian organ culture and was used to produce preantral follicles, in the second step oocyte–granulosa cell complexes were isolated from the ovarian organ and further cultured to complete oocyte growth and development (Figure 1a). In order to reconstruct the entire mouse oogenesis process *in vitro* from endogenous PGCs or PGCLCs obtained from ES or iPS cells, numerous exploratory experiments were implemented (Table 1). In 2002 Obata *et al.*^12^ translocated the nucleus of IVG oocytes that were incapable of resuming meiosis into completely growth oocytes of the normal adult mouse, then the reconstructed oocytes were subjected to IVM and fertilization and resulted in live offspring (Figure 1b). In 2016 a multistep culture procedure was devised by Morohaku *et al.*^2^ and Hikabe *et al.*,^3^ respectively (Figures 1c and 2). Morohaku and colleagues significantly improved the procedures to obtain secondary follicles from intact embryonic ovary cultures and to produce oocytes able to complete meiotic maturation and support embryo development from cultured oocyte–granulosa cell complexes. Hikabe and colleagues following Morohaku *et al.* added two steps to the procedures consisting of producing PGCLCs from epiblast like cells (EpiLCs) and reconstructing embryonic ovaries by aggregating PGCLCs with ovarian somatic cells (Figure 2). In this way the authors were able to, for the first time, reconstitute *in vitro* the entire cycle of the mouse female germ line from embryo to adult. Essentially, these authors combined previously developed procedures and used information obtained by other groups to reproduce, *in vitro,* distinct stages of oogenesis (Tables 1 and 2).

## PGCLCs Induction from EpiLCs *In Vitro* and their Development into Germ Cells *In Vivo*

Culture systems in which PGC specification processes were reconstituted *in vitro* from the epiblast had been developed in the early 2000s.^13, 14, 15, 16^ More recently, PGCLCs were obtained from ESCs and iPSCs.^17, 18, 19^ In such systems, the ESCs/iPSCs were first differentiated into a novel type of cell resembling the post-implantation epiblast called EpiLCs by inducing this status under defined conditions using basic fibroblast growth factor (bFGF) and activin A (ActA). The EpiLCs then efficiently differentiated into PGCLCs in response to BMP4, Kit ligand (KL), leukaemia inhibitory factor (LIF) and epidermal growth factor (EGF). Such PGCLCs were shown to be fully functional, if they were moved from *in vitro* to *in vivo* conditions, and appeared capable to differentiate into fertile sperms or mature oocytes; in the first case, upon *in vivo* transplantation into testicular tubules, in the second, after aggregation with 12.5 dpc ovarian somatic cells to reconstitute an ovary and then transplanting under the ovarian bursa of immunocompromised mice.^19^ In both cases, eggs fertilized with PGCLC-derived sperms or eggs-derived from PGCLCs fertilized by sperms from a donor male were showed to be able to give rise to healthy pups that eventually developed to fertile adults. PGCLCs were obtained from epiblast stem cells (EpiSCs) by Hikabe and colleagues using basically the same procedure described above.^3, 19^

## Reconstituted Embryonic Ovaries with PGCs and Ovarian Somatic Cells

Although cultures of isolated PGCs allowed the identification of factors crucial for their survival, proliferation and differentiation such as KL, LIF, bFGF and retinoic acid,^20^ they also showed that germ cells were unable to progress beyond the first stage of meiotic prophase I outside the gonadal microenvironment. Likewise, mouse fetal oocytes stimulated by KL appeared able to begin the growing phase in the absence of somatic cell support but needed follicular cells to further progress through development.^21^ For this reason, in an attempt to achieve complete oogenesis *in vitro*, various germ cell-ovarian somatic cell aggregation methods were utilized. Early experiments had shown that aggregation of PGCs with gonadal somatic cells resulted in reconstructed gonads that could be cultured *in vitro* for a short time to study some aspects of their proliferation and differentiation or transplanted *in vivo* to achieve complete gametogenesis. In particular, a number of papers showed that ovaries reconstructed by aggregating pre-meiotic female PGCs with ovarian somatic cells and transplanting under the kidney capsule or the ovarian bursa of syngeneic hosts were able to form preantral and antral follicles and develop into meiotic competent and fertilizable oocytes.^22, 23, 24, 25^ In this regard, it is important to point out that the developmental stage of the ovary from which PGCs were isolated and the proper synchronization of the germ cell–somatic cell interactions were considered crucial for germ cell survival and completion of oogenesis after transplantation. In fact, Lei *et al.*^22^ found that in transplanted reconstructed ovaries before 13.5 dpc, PGCs completed prophase of meiosis I but did not survive and form follicles. The same results were obtained when 12.5 dpc PGCs were aggregated with somatic cells from fetal ovaries of later developmental stages, competent to form follicles after kidney capsule transplantation or *in vitro* culture.^25^ In contrast, other papers, reported that aggregates of 12.5 dpc ovarian cells following heterotopic transplanted into adult females were able to produce fertilizable oocytes.^23, 25^

## Culture of Reconstituted Ovaries

Female mouse PGCs isolated and aggregated with ovarian somatic cells of the same or other stages were found to enter meiosis and give rise to primary oocytes of 30–35 *μ*m diameter. These cells appeared, however, unable to form follicles or to further progress into the growing phase under completely *in vitro* conditions.^22, 26, 27^

**Figure 3:**
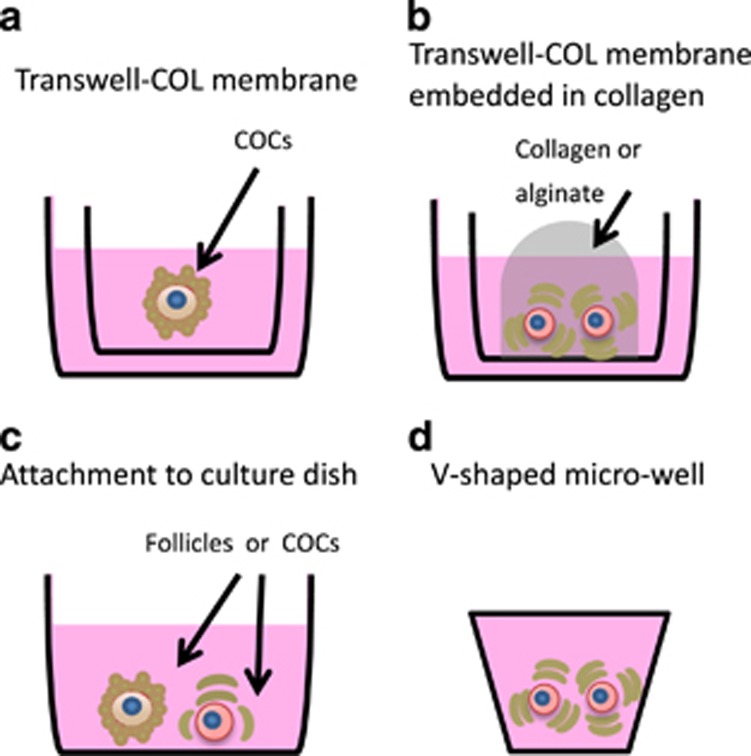
Schematic representation of four culture methods developed for mouse and human primordial/primary follicles or oocytes–cumulus cell complexes (COCs). (**a**) Eppig and O’Brien;^11^ O’Brien *et al.*^39^ (mouse). (**b**) Pangas *et al.*^71^; Mochida *et al.*^42^ (mouse). (**c**) Shen *et al.;*^43^ Cortvrindt *et al.*^44^ (**d**) Telfer *et al.* (human)^72, 73^

As far as we know, before the Hikabe and colleagues’ paper,^3^ the only attempt to culture PGCLCs *in vitro* within ovarian somatic cell aggregates was that by Hayashi and Surani.^28^ In this work PGCLCs obtained from EpiSCs were mixed with gonadal cells from 12.5 dpc ovaries and, after centrifugation, the cell suspension was left to aggregate and seeded through the gaps present between small pieces of 12.5 dpc ovaries on transwell inserts (Figure 3a). After 40 days in culture, oocyte-like cells (OLCs) probably derived from PGCLCs were observed although with a very low frequency of around one OLC out of 5000 PGCLCs. Conversely, Hikabe and colleagues aggregated PGCLCs with 12.5 dpc ovaries in a low-binding U-bottom 96-well plate (Figures 2 and 3d) for 2 days in serum-free GK15 with knockout serum replacer and the average number of germinal vesicle (GV) stage oocytes per recombinant ovary (consisting of 5000 PGCLCs and 50 000 gonadal somatic cells) was 55.1.^3^

The conditions for long-term *in vitro* culture of reconstructed mouse embryonic ovaries suitable to obtain secondary follicles containing mature GV stage oocytes capable of supporting embryo development after fertilization were not established until the paper by Hikabe *et al.*^3^ This method was established on the basis of the results obtained in a parallel paper in which the same group was able to reconstruct complete oogenesis *in vitro* from 12.5 dpc whole ovaries.^2^

Before the Morohaku and colleagues’ work,^2^ several attempts were performed in the mouse to produce fertilizable oocytes entirely *in vitro* by culturing intact or pieces of embryonic ovaries (Figure 1a and b and Table 1). The more advanced developmental phases of mouse oogenesis obtained *in vitro* by culturing intact embryonic ovaries was the preantral and antral follicle stages^12, 29, 30^ (Figure 1b and Table 1). However, GV stage oocytes obtained from such follicles were unable to resume meiosis. Other authors cultured whole or pieces of embryonic ovaries allowing them to attach and spread onto the bottom of the culture dish (Figure 3c).^27, 31, 32, 33, 34, 35^ In such cultures, mouse PGCs were able to differentiate into primary oocytes that were eventually assembled into primordial or primary follicles. Within these structures, apparently morphologically normal GV stage oocytes grew considerably in volume reaching a maximum diameter of around 65–70 *μ*m, but did not acquire the capability to resume and complete meiosis. The only exception was reported by Shen’s group,^35^ who showed that a small number of such oocytes were able to resume meiosis and be fertilized providing ActA was present in the medium throughout the entire culture time (Table 1).

These experiments pointed out that it was relatively simple to induce the development of female PGCs into primary oocytes that are able to complete meiotic prophase I and be assembled into primordial/primary follicles and almost complete the growing phase inside cultured ovarian tissues *in vitro*. Moreover, this system allowed the identification of some key factors necessary for oocyte and/or granulosa cell development such as KL, EGF, IGF-1, Activins and others (Table 2). However, they also indicated that almost invariably such oocytes lacked factors necessary to acquire complete meiotic maturation and the capacity to support embryo developmental following fertilization.

## Oestrogen Inhibition Favours PF Assembly While Polyvinylpyrrolidone Stabilizes Correct Oocyte–Granulosa Cell Relationships in the Secondary Follicles

In our view, a major breakthrough of the Morohaku and colleagues studies was to establish *in vitro* culture conditions that allowed more efficient formation of PFs within the cultured ovaries and the maintenance of the correct interactions between the oocyte and the granulosa cells during the subsequent development of the primary and secondary follicles.^2^ They achieved such important points apparently in a very simple way, by timely adding to the culture medium of ovarian tissues an oestrogen receptor antagonist (ICI 182,780, 7*α*,17*β*-[9-[(4,4,5,5,5-Pentafluoropentyl)sulfinyl]nonyl]estra-1,3,5(10)-triene-3,17-diol) and a high concentration (2%) of polyvinylpyrrolidone (PVP), a water soluble polymer made from the monomer *N*-vinylpyrrolidone.

The reason for adding ICI was that previous papers clearly showed that reduced concentrations of oestrogens and/or progesterone were essential to promote germ cell cyst breakdown and follicle formation in mice and rats.^36, 37^ As a matter of fact, oestrogens are present in the fetal calf serum (FCS), necessary for optimizing the ovary culture, and likely produced by the cultured granulosa cells. Morohaku and colleagues described that the addition of ICI from day 5 to day 11 of culture of the 12.5 dpc ovary (equivalent to 17.5 dpc – 4 dpp, the period of PF assembly in the mouse), resulted in a more than sevenfold increase in the number of secondary follicles isolated from the cultured ovaries on day 17 of culture. Moreover, ICI treatment promoted the formation of a complete laminin layer around individual follicles.

Secondary follicles were mechanically isolated on day 17 of culture since longer culture of the whole ovaries resulted in marked follicle degeneration. These follicles, containing oocytes with a mean diameter of 54.4 *μ*m, were then cultured for 3 days in minimum essential medium-alpha supplemented with 5% FCS, 0.1 IU FSH and 2% PVP on a millicell or transwell-coll membrane. In addition, Hikabe and colleagues added BMP15 and growth differentiation factors 9 (GDF9), two growth factors secreted by the oocytes to stimulate granulosa cell proliferation and steroidogenesis.

The exact role of PVP in this culture step, as well as in the next (see below), is not known. PVP and polyvinylalcohol are frequently added at low concentration (not >0.4%, w/v) to medium as a substitute for macromolecules in serum, which provide some colloid osmotic pressure and are useful for preventing loss of oocytes/embryos due to sticking to the glass or plastic surface. Morohaku and colleagues suggest that they add PVP because of its effect on maintaining the integrity of oocytes–cumulus cell complexes (COCs) in a long-term culture found in their previous studies in cattle.^2, 38^ In addition to its notable effect on favouring correct oocyte–granulosa cell growth in the secondary follicles, PVP was found to positively affect the expression of genes encoding factors involved in granulosa cell proliferation, such as *Bmp6*, *Bmp15*, *Kit* and *Kitl*. A plausible explanation of the PVP effect is that it and other beneficial factors were localized within the complexes due to restricted diffusion.

## Removal of Follicular Theca and the Presence of PVP are Essential for Complete COC Maturation

To reconstitute gametogenesis under complete *in vitro* conditions, Morohaku *et al.*^2^ recently established a robust protocol to faithfully give rise to viable offspring. Female gonads obtained at 12.5 dpc were cultured for 17 days on transwell-collagen (COL) membranes, after the initial culture for 3 days, the resulting secondary follicles were treated with collagenase to remove theca layers and allow the oocytes to undergo full growth, maturation, fertilization and complete embryo development (Figure 2). It is probable that exposure of COCs directly to the culture medium may promote oocyte development by supporting direct exchange of compounds. Theca removal had been first used to culture mouse COCs in a two-step culture protocol devised by the Eppig group. The result of this system was the production of oocytes competent to undergo maturation, fertilization and development to live offspring starting from the PFs of newborn mice.^11, 39^ Briefly, ovaries were initially isolated from newborn mice and cultured on Millicell-PC membrane inserts (organ culture), for the second step the cultured ovaries were digested with collagenase and DNase to isolate oocyte–granulosa cell complexes and these complexes were cultured for 8 days on transwell-COL membranes (follicle culture).

Subsequent COCs were cultured for another 9–13 days under the same conditions reported above resembling that of Eppig and O’Brien^11^ and O’Brien *et al.,*^39^ but with two main differences: the presence of 5% FCS and 2% PVP. At this stage, PVP appeared important for maintaining compact COCs attached to the insert membrane allowing a significantly larger number of complexes to be recovered. Although a direct comparison of the protocols has not been studied, it was likely that in the Eppig and O’Brien protocols the function of FCS and PVP was replaced, at least partially, by BSA, fetuin, EGF and ITS. The possibility to achieve functional oocytes within COCs independent from the formation of antral follicles is a really important point. It provides a solution to the completion of folliculogenesis *in vitro,* in species producing large preovulatory antral follicles such as humans, which is a problem for this type of culture system. Utilizing these novel culture conditions, the potential problems of nutrient, gas and waste exchange are considerably reduced. As we will discuss below, unlike the antral follicle size, the size and structure of COCs vary little among mammalian species and conditions required for maturation of such complexes *in vitro* are likely to be quite similar.

The subsequent steps of COC mucification and oocyte maturation to the metaphase II stage and finally IVF and embryo transplantation were performed according to standard procedures.^11, 39^

## Developmental Competence of Oocytes Grown *In Vitro* Under Different Experimental Protocols

Although the papers by Morohaku *et al.* and Hikabe *et al.* are so far the only that show the complete reconstituted *in vitro* mouse oogenesis, it is useful to compare their results to those of the Eppig’s group. Their groundbreaking work was the first to report the possibility of producing functional mouse oocytes from PFs capable of giving rise to live offspring after fertilization, entirely *in vitro*. Actually, it is likely that Morohaku and colleagues and Hikabe and colleagues referred to these works for development of their COC culture system.

By comparing the results obtained by Morohaku *et al.* and O’Brien *et al.* (Table 3), it clearly appears that the major difference is in the percentage of oocytes able to mature to the metaphase II (Met II) stage within COCs after FSH and LH stimulation. Interestingly, the competence of the mature Met II oocytes to be fertilized and give rise to live offspring are quite similar. Since the protocol of COC culture is almost the same, and O’Brien and colleagues used neonatal ovary developed *in vivo*, the reason for such a difference must be in some critical aspect of oocyte development, performed *in vitro* in both cases, when PF are assembled and the folliculogenesis is activated up to the secondary follicle stage. In fact, O’Brien and colleagues cultured for 8 days newborn ovaries basically only in Waymouth medium supplemented with 10% FCS, while Morohaku and colleagues added the oestrogen receptor antagonist (ICI182780) and PVP, likely indispensable to more closely recapitulate the physiological ovary development at this stage. However, in the Hikabe paper, the same protocol used in Morohaku and colleagues appears much less efficient in producing mature oocytes and pups when applied to PGCLCs, which may be caused by the deficiency of the current PGCLCs differentiation system, either at the transcriptional or epigenetic level.

In all these works, the timing of the *in vitro* culture parallels that of mouse oogenesis characterized by a prenatal development of 18–19 days and a first wave of folliculogenesis starting around birth and producing antral follicles after about 3 weeks. All procedures were of course able to produce only one wave of mature oocytes since the cyclic nature of oogenesis could not be reproduced using such systems. Noteworthy, Morohaku and colleagues reported that a maximum of seven pups were obtained from a single gonad, which is comparable with the number seen in a natural cycle in mice.

## Prospects

The procedures discussed above developed in the mouse raise the possibility of growing human oocytes to maturity using a similar *in vitro* culture system. The potential of such a system with regard to improving human fertility would be great. It would clearly be of benefit in basic physiological studies on human oogenesis. It could be used to test the effect of toxicological substances on oocyte and follicle maturation. Eventually, such a system could provide a source of oocytes for ARTs. Noteworthy, Morohaku and colleagues reported that their procedure was able to produce functional oocytes also from cryopreserved fetal mouse gonads, a common practise in ARTs.

The potential to generate human PGCLCs from ES and iPS cells^40^ then growing functional oocytes,^41^ using procedures quite similar to that used in the mouse is probable due to the fact that the size and structure of COCs vary little among mammalian species. It is likely that the systems will have to be modified for culturing complexes from species other than mouse, in particular to restrict the migration of granulosa cells away from the oocytes, a problem apparently solved in mouse and bovine by PVP. The maintenance of the association between granulosa cells and the oocyte is essential for oocyte growth and development. On the contrary, the apparent asynchrony of PGC/oogonia proliferation and entering into meiosis together with a lack of information about how these processes are regulated in the human fetal ovary, and the scant knowledge of the mechanisms of PF assembly and timing of transition from primordial to primary and secondary follicle stage represent critical points. In the case of using PGCLCs, another limitation is the requirement of gonadal somatic cells for reconstructing the embryonic ovary. As suggested by Hikabe and colleagues to overcome this issue gonadal somatic cell-like cells could be derived from ES or iPS cells. This is a critical step that will need to be addressed to ethically utilize a similar system for human reproduction.

For this reason, the first step towards adapting a culture system for use in humans is to acquire more information about the critical points reported above, and at the same time use animal models with oogenesis characteristics more similar to humans and with an abundant supply of materials. Repeating these studies in non-human primates would be beneficial in elucidating the likelihood of transitioning the findings to humans.

## Figures and Tables

**Table 1:** Culture systems devised in the attempt to reconstitute various stages of mouse oogenesis *in vitro*

**Germ cell sources**	**Culture method**	**Results**	**Year**	**References**
12.5 dpc ovary	OC attached on plates	MII	2009	^29^
12.5 dpc ovary	OC Transwell membrane on+FC on Transwell-COL membrane+NT	Pups	2002	^12^
12.5 dpc ovary	OC on Transwell-COL membranes+FC on Millicell membrane+FC on Transwell-COL or Millicell membranes	Pups	2016	^2^
16.5 dpc ovary	OC on Millicell-PC membrane+ FC culture in droplets	Morulae-blastocysts	2007	^45^
Primordial follicles	OC on Millicell-PC membrane+FC Transwell-COL membrane	Pups	1996, 2003	^11, 39^
Primary/early secondary follicles	FC in Collagen gels+ on Transwell-COL membrane	Pups	2013	^42^
Oocytes from 12–14 dpp ovary	Oocytes co-cultured with PAGCs	Morula -blastocysts	2011	^46^
ES cells	Reconstituted ovaries+transplantation	Offspring	2012	^17^
ES cells	Reconstituted ovaries+IVDi +IVG	Offspring	2016	^3^

Abbreviations: COL, collagen; FC, follicles culture; IVDi, *in vitro* differentiation; IVG, *in vitro* growth; MII, meiosis II; NT, nuclear transfer; OC, organ culture; PAGCs, preantral granulosa cell

**Table 2:** Some key factors associated with the development of mouse oocyte and follicle *in vitro*

**Factor/s**	**Culture method**	**References**
FSH	Essential for follicular survival and development, contributes to granulosa cells proliferation	^44, 47, 48, 49, 50, 51^
EGF and IGF-I	Have an effect on follicle growth and development and embryo development after fertilization.	^11, 52, 53^
Activins	Promote antral cavity formation and granulosa cells proliferation, affect follicle growth and development	^35, 54, 55, 56, 57, 58, 59, 60^
KL (or SCF)	Promote the oocyte survival and growth	^21^
cAMP	Control of meiotic progression, gap–junctional communication between granulosa cells and oocyte and antral cavity formation	^61, 62, 63, 64, 65, 66^
PVP	Maintains the integrity of follicles or COCs in culture	^67, 68^
Ascorbic acid	Acts likely as antioxidant preventing deleterious action of the free radicals	^69, 70^
Collagen	Favours follicle/COC adhesion, stimulates cell proliferation and development and support biological signalling pathways	^3, 11, 42, 43^
ICI182780	Antagonist of oestrogen receptor, its addition to the culture medium of ovarian tissue results in a marked increase of secondary follicle number	^2, 3^
Theca layers	Their removal exposes COC to the culture medium resulting in considerable improvement of its development and the acquisition of meiotic maturation by the oocyte	^2, 3^

**Table 3:** A comparison of different protocols used to produce functional oocytes

	**Morohaku** ***et al.***^67^	**Hikabe** ***et al.***^3^	**O’Brien** ***et al.***^39^
Met II	84 (90)	28.9 (−)	44 (>90)
2-cells	43.5 (48.2)	36 (−)	43 (63)
Pups	27 (59.7)	3.5 (61.7)	5 (16.5)

Values represent % in parenthesis the values of control *in vivo* matured oocytes; ‘−’ means no available data
